# Spatiotemporal genomic patterns of *Quercus gilva*: decoupling historical isolation from contemporary environmental adaptation

**DOI:** 10.48130/forres-0026-0016

**Published:** 2026-04-28

**Authors:** Shan-Shan Wang, Tian-Rui Wang, Ling-Ling Wang, Pei-Han Huang, Si-Di Liang, Si-Si Zheng, Liang-Hai Yang, Yu Li, Hong-Hu Meng, Xin Zhong, Xiao-Chen Li, Bin-Jie Ge, Zi-Jia Lu, Yi-Xin Kang, Quan Yuan, Dong-Mei Jin, Xin Ning, Gregor Kozlowski, Young-Jong Jang, Jin Xu, Jung-Hyun Lee, Yi-Gang Song

**Affiliations:** 1 Key Laboratory of East China Plant Conservation and Utilization, National Forestry and Grassland Administration, Shanghai Chenshan Botanical Garden, Shanghai 201602, China; 2 School of Urban Construction and Ecological Technology, Shanghai Institute of Technology, Shanghai 201418, China; 3 Plant Phylogenetics and Conservation Group, Centre for Integrative Conservation, Xishuangbanna Tropical Botanical Garden, Chinese Academy of Sciences, Kunming 650223, China; 4 Department of Biology and Botanic Garden, University of Fribourg, Fribourg 1700, Switzerland; 5 Natural History Museum Fribourg, Fribourg 1700, Switzerland; 6 Department of Biology Education, Chonnam National University, Gwangju 61186, Republic of Korea; 7 College of Forestry and Biotechnology, Zhejiang A & F University, Hangzhou 311300, China

**Keywords:** Climate change, East Asia, Evolutionary trajectory, Population structure, *Quercus gilva*, Whole-genome resequencing

## Abstract

The interplay between historical biogeography and environmental selection shapes the genetic architecture of species; however, their relative contributions in widespread and ecologically important tree species remain poorly understood. In the Sino-Japanese Floristic Region, the East China Sea has alternately acted as a barrier and a corridor during glacial-interglacial cycles, influencing species isolation and contact. However, the extent to which these historical dynamics, along with environmental gradients, have driven adaptive evolution in dominant forest trees remains unresolved. In the present study, we integrated phylogeographic reconstruction and landscape genomics based on whole-genome resequencing data from 171 individuals across 35 populations to investigate the evolutionary history and adaptive mechanisms of *Quercus gilva*, a keystone evergreen oak, in East Asian subtropical forests. Population genomic analyses revealed two deeply divergent genetic groups: the China group and the Japan–Korea group, and demographic modeling indicated that their divergence dates back to the Miocene epoch. The relatively high genomic diversity of *Q. gilva* may result from the absence of severe genetic bottlenecks throughout its evolutionary history, multiple microrefugia during the glacial periods, and postglacial secondary contact. Notably, Quaternary glacial cycles repeatedly facilitated gene flow and admixture between these groups via exposed land bridges. Landscape genomic analyses further demonstrated that adaptive divergence in *Q. gilva* is shaped by both geographic isolation and environmental gradients, with annual precipitation emerging as the primary climatic driver. This study provides a genome-wide perspective on how historical biogeography and environmental selection interact to shape the genetic architecture of a dominant subtropical evergreen forest species, offering novel insights into the evolutionary dynamics and adaptive evolution across East Asian biodiversity hotspots.

## Introduction

Understanding evolutionary processes across spatiotemporal scales in a local context is critical for biodiversity conservation^[1,2]^. Populations are the fundamental units of evolution and play a crucial role in maintaining the genetic diversity of species, environmental adaptation, and ecosystem restoration^[3]^. To avoid local extinctions, populations must either track suitable habitats or adapt to new environmental conditions through the fixation of standing genetic variation and *de novo* mutations^[4−8]^. Thus, dissecting evolutionary patterns through population genetics, demography, and geography helps us understand how genetic variation is distributed across landscapes and the evolutionary potential of species under future climate change^[9−12]^. The recent development of high-throughput genomic tools has further enabled a shift from traditional phylogenetics and landscape genetics toward phylogenomics and landscape genomics.

Species have persisted through repeated periods of fluctuating climate, such as the Quaternary ice ages, which caused major changes in global sea levels and the extent of continental ice sheets^[13]^. Because of their sedentary lifestyle, long lifespans, and slow generation times, tree species often display geographic patterns of genetic variation largely shaped by local adaptation^[10−12]^. East Asia remained largely ice-free during the ice ages, serving as a refugium for many woody plant taxa^[14−17]^. The Sino-Japanese Floristic Region (SJFR), the core of East Asian subtropical evergreen broad-leaved forests (EBLFs), is a global hotspot of woody plant diversity with complex topography and relatively little impact from Quaternary glacial-interglacial cycles^[17−20]^.

Understanding the evolutionary history of EBLFs is essential not only for biodiversity conservation but also for predicting their responses to future climate change^[10−12,17,21−24]^. Over the past two decades, phylogeographic studies using first-generation sequencing have examined various EBLF species^[19,20,25−29]^. More recently, population genomic analyses using next-generation sequencing have provided deeper insights into the evolutionary history and adaptive mechanisms of these species^[10−12,23,24,30,31]^. Collectively, these studies reveal strong phylogeographic patterns shaped by monsoon evolution, Tibetan Plateau uplift, and geographic barriers^[19]^. However, genomic insights into the evolutionary processes across spatiotemporal scales and the potential vulnerability of EBLFs under future climate change remain limited.

*Quercus gilva* is an endangered evergreen tree species with high ecological and cultural value^[32]^. It occurs at low to middle altitudes, where it is heavily impacted by human activities. As a result, present-day populations of *Q. gilva* are mostly restricted to fengshui forests (e.g., around temples and shrines) and consist of only small numbers of individuals. Although widely distributed across the SJFR, its populations are sparse, small, and exhibit a scattered distribution^[32−34]^. Recently, the genome of *Q. gilva* was published, facilitating studies on the ecological adaptation and evolution of oaks^[35]^. Analyses of genetic diversity and population structure based on genome-wide variation have been conducted, but only on limited samples^[32]^. Consequently, the roles of environmental and geographic gradients in shaping population differentiation, as well as the potential responses of *Q. gilva* to future climate change, remain poorly understood.

To address these gaps, we conducted a phylogeographic and landscape genomics study of *Q. gilva* using whole-genome resequencing data from the most comprehensively sampled natural populations of the species. Using genome-wide genetic variation, we inferred the spatial patterns of genetic diversity, population structure, and evolutionary trajectories of *Q. gilva*. We also quantified the contributions of environmental and geographic factors to these spatial genetic patterns. Finally, we evaluated the genomic vulnerability of different genetic groups under future climate scenarios. This study enhances our understanding of the spatiotemporal evolution of *Q. gilva* and informs conservation strategies for tree species in EBLFs under climate change.

## Materials and methods

### Sample collection and whole-genome resequencing

We sampled 171 individuals from 35 natural populations spanning the entire distribution range of *Q. gilva*, including populations from China, Japan, and South Korea (Supplementary Fig. S1, Supplementary Table S1). For each sample, genomic DNA was extracted from mature leaves using the standard cetyltrimethylammonium bromide (CTAB) method^[36]^. DNA quality and concentration were assessed using 0.75% agarose gel electrophoresis, NanoDrop One spectrophotometer (Thermo Fisher Scientific), and Qubit 3.0 fluorometer (Life Technologies, Carlsbad, CA, USA). Sequencing libraries were constructed according to the manufacturer's instructions, followed by sequencing using combinatorial probe anchor synthesis (cPAS) technology^[37]^. Paired-end sequencing (150 bp) was performed on a DNBSEQ-based platform (DNBSEQ-T7) using a commercial service (Wuhan Benagen Tech Solutions Company Limited, Wuhan, China).

### Genome mapping and single-nucleotide polymorphism (SNP) calling

Adapter trimming and read filtering were performed using Fastp^[38]^ to obtain high-quality, clean paired-end reads. The specific steps included: removal of adapter sequences, trimming of trailing polyG/polyX sequences (minimum length: 10 bp), and discarding of reads containing more than 5 'N' bases, with an average quality score below 20, or more than 40% of bases having a quality score below 15. Finally, reads shorter than 50 bp were excluded. The cleaned reads were aligned to the *Q. gilva* reference genome^[35]^ using BWA^[39]^ software with default parameters to generate the BAM files. The resulting files were sorted using SAMtools v1.9^[40]^ and duplicates were removed using Picard v2.18.11 (http://broadinstitute.github.io/picard). Genetic variant calling was performed using the Genome Analysis Toolkit (GATK v4.3.0.0) pipeline, employing the HaplotypeCaller, CombineGVCFs, and GenotypeGVCFs tools to generate a consolidated VCF file. After the initial variant calling, we used the GATK GenotypeGVCFs tool with the -includeNonVariantSites parameter to call variants and generate two datasets: one including 894,778,865 nonvariant sites, and the other containing 150,200,828 variant sites only. For the dataset that included nonvariant sites, filtering was performed using VCFtools v0.1.13^[41]^, retaining only biallelic sites with a minimum sequencing depth of 5 (--minDP 5) and a maximum missing rate of 20% (--max-missing 0.8). After filtering, 139,937,053 sites were retained for the estimation of nucleotide diversity (*π*) and for demographic history analyses. For the dataset excluding nonvariant sites, variant filtering was performed using the VariantFiltration tool with the following stringent criteria: QD < 2.0 || FS > 60.0 || QUAL < 30.0 || SOR > 3.0 || MQ < 40.0 || MQRankSum < −12.5 || ReadPosRankSum < −8.0, to filter the identified SNPs^[42]^. Additional filtering was conducted using VCFtools v0.1.13^[41]^ with the following parameters^[11]^: (1) --minDP 5, --maxDP 50, --min-alleles 2, --max-alleles 2, --max-missing 0.8, --maf 0.05, resulting in 17,396,250 SNPs (Dataset 1); (2) the same parameters applied to a dataset including five outgroups, resulting in 15,795,567 SNPs (Dataset 2); and (3) --minDP 5, --maxDP 50, --min-alleles 2, --max-alleles 2, --max-missing 0.95, --maf 0.05, resulting in 11,762,059 SNPs (Dataset 3).

### Population structure and phylogenetic analysis

We performed linkage disequilibrium (LD) pruning on Datasets 1, 2, and 3 using PLINK v1.90^[43]^ with the following parameters: -indep-pairwise 50 10 0.2. Dateset 1 yielded 2,934,638 independent SNPs for population genomic analysis (including ADMIXTURE, principal component analysis [PCA], genetic diversity, differentiation, inbreeding, demographic history, and gene flow), and 1,026,841 independent SNPs were yieled from Dateset 3 for landscape genomic analysis (including Mantel tests, redundancy analysis [RDA], Gradient Forest [GF] analysis, BayeScan, latent factor mixed modelling [LFMM], and risk of non-adaptedness analysis [RONA])^[44]^. Population structure analysis was inferred using ADMIXTURE v1.3.0^[45]^, with the number of ancestral clusters (*K*) tested from 1 to 10. The optimal *K* value was determined based on the lowest cross-validation error. To further explore the genetic relationships among individuals, PCA was conducted in PLINK^[46,47]^ using the LD-pruned SNP dataset. The resulting principal components (PCs) were visualized using the first two axes (PC1 and PC2) in R v4.2.2, implemented with the 'FactoMineR'^[48]^, 'factoextra'^[49]^, and 'ggplot2'^[50]^ packages.

Phylogenetic reconstruction based on Dateset 2 was performed using the Maximum Likelihood (ML) method in IQ-TREE v1.6.12^[51]^. The SNP dataset was first converted from the VCF to the PHYLIP format (.phy) using the vcf2phylip.py script. The optimal nucleotide substitution model ("GTR + F + ASC + R6") was selected from 352 candidate models using ModelFinder^[52]^ under the Bayesian Information Criterion. Branch support was evaluated using 1,000 ultrafast bootstrap replicates. The maximum likelihood tree was rooted using five outgroup species: *Quercus robur*, *Q. acutissima*, *Q. pachyloma*, *Q. augustinii*, and *Q. rex* (Data from unpublished work of our group). The final phylogenetic tree was visualized using FigTree v1.4.4 (http://tree.bio.ed.ac.uk/software/figtree)^[53]^.

Divergence time estimation was conducted using Bayesian Markov Chain Monte Carlo (MCMC) algorithms implemented in BEAST v1.10. The best-fit substitution model ("GTR + F + ASC + R6") was selected using the ModelFinder^[52]^. The Bayesian inference tree was calibrated using three fossil constraints derived from Fagaceae phylogeny: genus *Quercus* (60.52–55.18 Ma)^[54]^, section *Cyclobalanopsis* (48.98–47.70 Ma)^[55]^, and the divergence between *Q. jenseniana* and *Q. gilva* (24.80–17.87 Ma)^[54]^. The MCMC chain was run with the following settings: a burn-in of 1,000,000 generations, sampling every 10 generations, and a total of 'pf' 500,000 samples. The final phylogenetic tree was visualized and edited using FigTree v1.4.4.

### Genetic diversity, differentiation, and inbreeding analyses

To assess genome-wide patterns of genetic diversity and differentiation, we calculated *π*, inter-population divergence (*D*_XY_), genetic differentiation (*F*_ST_), and Tajima's *D* in 100-kb sliding windows using pixy v1.2.7^[56]^ for all loci (including non-polymorphic loci). To ensure robustness, stringent filtering was applied: genomic windows with effective site coverage (including both polymorphic and monomorphic sites) below 10% were excluded from *π* and *D*_XY_ calculations, and windows containing fewer than 20 SNPs were omitted from the *F*_ST_ estimation. We then used PopLDdecay v3.4.1 to calculate the LD decay within each group and compared the patterns between the two groups^[57]^.

Runs of homozygosity (ROH) were identified for each individual using PLINK v2.0^[46]^ with the following stringent parameters: --homozyg-kb 10, --homozyg-snp 50, --homozyg-density 50, --homozyg-window-snp 20, with ≤ 1 heterozygous and ≤ 5 missing calls, --homozyg-het 1, --homozyg-missing 5. The inbreeding coefficient (*F*_ROH_) was calculated as the proportion of the genome contained within ROH regions, using the formula of *F*_ROH_ = $ \dfrac{\mathrm{∑}{\mathrm{L}}_{{ROH}}}{\mathrm{Lauto}} $, where '∑L*_ROH_*' represents the total length (in base pairs) of all ROH segments across the autosomes, and 'Lauto' represents the total physical length (in base pairs) of the autosomes covered by SNPs. Visualization of *F*_ROH_ distribution across individuals and populations was performed using the ggplot2 package in R.

### Population history inference and gene flow analysis

Two coalescence-based methods were employed to reconstruct the population demographic history of *Q. gilva*. First, we inferred changes in the effective population size over time using SMC++ v1.15.4^[58]^, which employs a coalescent hidden Markov model. This method uses all genomic sites to estimate historical population size changes for the China and Japan–Korea groups. The results were scaled to real time using a generation time of 50 years, and a mutation rate of 1.01 × 10^−8^ per site per generation. To further validate the results and infer long-term population dynamics, we applied the Pairwise Sequentially Markovian Coalescent (PSMC) model using consensus sequences generated from the BAM alignment files of all individuals. The model was run with the parameter setting "4 + 25 * 2 + 4 + 6" and similarly scaled using the same generation time and mutation rate.

To investigate historical gene flow among the major genetic clusters, we used TreeMix v1.13^[59]^ to infer population splits and migration events. We tested models allowing between one and ten migration edges, calibrated on the maximum likelihood tree. To avoid overfitting, the -noss option was applied. To determine the optimal number of migration events, we used the OptM package in R for model selection^[60]^. For the selected optimal m value, we calculated the proportion of variance explained using the R script treemixVarianceExplained.R. The final tree was visualized in R. Additionally, to quantify introgression signals, we calculated the D-statistic (ABBA-BABA test) using Dsuite v0.3^[61]^. We performed two separate analyses with the Dtrios function: one based solely on population assignments, and the other incorporating a known species tree to account for phylogenetic uncertainty. The results of both analyses were visualized as heat maps using the pheatmap package in R.

### Correlation between genetic and environmental variation

We assessed the influence of spatial and environmental factors on the genetic differentiation in *Q. gilva* using Mantel tests and RDA. Pairwise *F*_ST_ values were calculated with VCFtools and transformed to genetic distances using the formula *F*_ST_/(1 − *F*_ST_) in the vegan R package^[62]^. Nineteen bioclimatic variables were downloaded from WorldClim and filtered for multicollinearity (|r| > 0.8), using the usdm R package^[63]^. Environmental and geographic distance matrices were generated using the stats and geosphere packages in R, respectively, and Mantel tests were performed with 9,999 permutations.

To further explore the environmental and spatial effects on genetic variation, we conducted RDA and partial RDA using the vegan package in R. SNP data were filtered and converted into individual-level allele frequency matrices, whereas the environmental predictors included six noncollinear bioclimatic variables and sampling coordinates. In partial RDA, geographic coordinates were treated as covariates to control for spatial autocorrelation. Model significance was evaluated via 999 permutations (*α* = 0.05). We also applied gradientForest and extendedForest^[64,65]^ to characterize allele frequency turnover along environmental gradients and to identify key climatic drivers. Genetic input consisted of LFMM-format allele frequency data derived from filtered VCF files, and environmental input included sampling coordinates and six selected bioclimatic variables. GF analysis was performed using the R package with the following parameters: transform = NULL, compact = TRUE, nbin = 201, correlation threshold of 0.5 for predictor variables, and compact mode to optimize memory usage. The analysis assessed the contribution of each climatic variable to allelic variation, with results visualized using overall and cumulative importance plots.

### Detection of selection signatures and adaptive loci

We investigated the genomic basis of climate adaptation using two complementary approaches: BayeScan^[66,67]^ for detecting loci under diversifying selection and LFMM^[68]^ to identify genotype–environment associations. For the BayeScan analysis, we converted the VCF files into the required format using the vcf2bayescan.pl script and ran the analysis with the following parameters: 20 pilot runs (5,000 iterations each), 50,000 burn-in steps, a thinning interval of 10, and a prior odds ratio of 100. Significant outlier loci were identified based on internally calculated *q*-values, which represent posterior probabilities corrected for the false discovery rate (FDR), using a threshold of *q* < 0.05. LFMM analysis was performed using the LEA package in R. Missing genotypes were first imputed via the 'snmf' function. Based on the inferred population structure (*K* = 2), 10 replicates of the LFMM model were run, each with 100,000 iterations and a burn-in of 50,000. To control for false positives due to multiple testing, we applied FDR correction, and SNPs with an adjusted *p*-value (*q*-value) < 0.05 were identified as significant outliers associated with environmental variables. Functional annotation of all variant sites was performed using snpEff^[69]^, and Gene Ontology (GO) enrichment analysis was conducted with AnnotationForge on two variant sets: (1) overlapping outliers identified by BayeScan and LFMM, and (2) missense variant SNPs.

### Predicting future adaptive potential and ecological niche modeling

We used RONA to quantify theoretical allele frequency shifts and infer the adaptive potential of *Q. gilva* under future climate scenarios. Individual-level allele frequencies were extracted using the LEA package^[70]^ and regressed against six uncorrelated bioclimatic variables. The predicted allele frequencies for 2081–2100 under the SSP126 and SSP585 scenarios were generated using these models, and the average deviation from the present-day frequencies was calculated as the RONA value. Higher RONA values indicate greater genetic vulnerability and reduced adaptive potential.

Species distribution modeling was conducted in MAXENT v3.4.4^[71]^ using occurrence records and 2.5-arcmin resolution bioclimatic data from WorldClim across seven time periods: Last Interglacial (LIG), Last Glacial Maximum (LGM), Mid-Holocene, present (1970–2000), and three future windows under SSP126/SSP585. We screened the environmental variables for collinearity using the vifcor function (R package usdm) with a Pearson correlation threshold of |r| > 0.8. Following this screening, six predictors were retained for subsequent analyses: bio5 (maximum temperature of the warmest month), bio8 (mean temperature of the wettest quarter), bio9 (mean temperature of the driest quarter), bio12 (annual precipitation), bio15 (precipitation seasonality), and bio17 (precipitation of the driest quarter). Model tuning was performed using ENMeval, testing 48 combinations of regularization multipliers and feature classes. The optimal model (ΔAICc = 0) was selected for final projections. Habitat suitability was classified in ArcGIS 10.2 based on logistic output into four categories: non-suitable (*p* < 0.2), low (0.2 ≤ *p* < 0.4), moderate (0.4 ≤ *p* < 0.6), and high suitability (*p* ≥ 0.6), following thresholds previously applied in *Quercus* sect. *Heterobalanus*^[72]^. The suitable area for each category was then quantified.

## Results

### Sequence data

We generated 2.999 Tb of clean sequencing data from 171 *Q. gilva* individuals (Supplementary Table S2). After quality filtering, three SNP datasets were obtained for downstream analyses: (1) Dataset 1: 2,934,638 SNPs for genetic structure analysis (filtered with --max-missing 0.8, retaining loci with ≤ 20% missing data across samples). (2) Dataset 2: 2,763,859 SNPs, including five outgroups (*Q. acutissima*, *Q. pachyloma*, *Q. rex*, *Q. augustinii* and *Q. lobata*) for phylogenetic analysis. (3) Dataset 3: 1,026,841 SNPs for landscape genomic analysis (filtered with --max-missing 0.95, retaining loci with ≤ 5% missing data across samples).

### Population structure of *Q. gilva*

**Figure 1:**
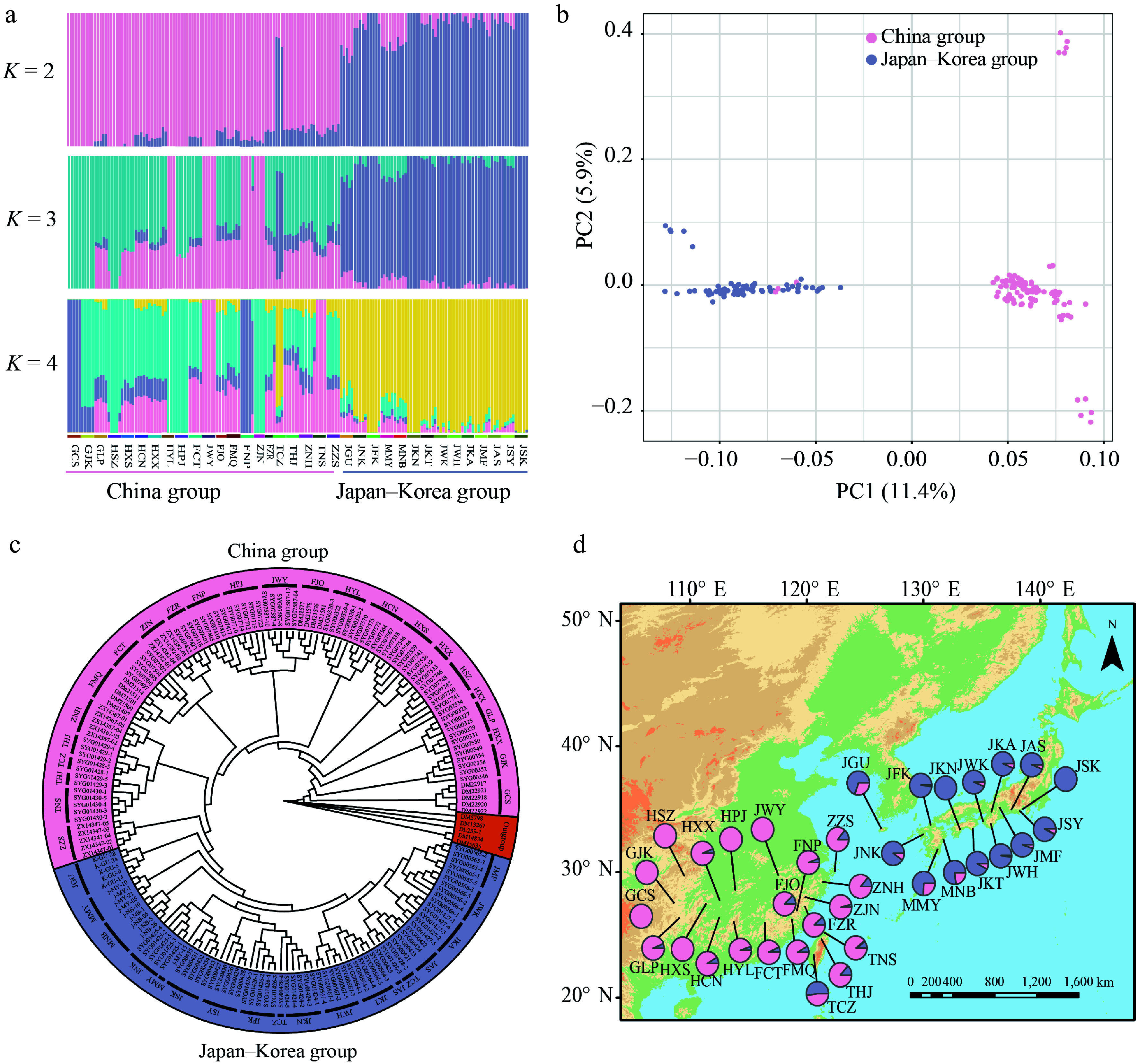
Geographic distribution and population genetic structure of *Quercus gilva*. (a) ADMIXTURE bar plots for *K* = 2, *K* = 3, and *K* = 4. The *x*-axis represents populations, and the *y*-axis shows the inferred ancestry proportions. (b) Principal component analysis (PCA) with color-coding to indicate different groups of *Q. gilva*. Light pink and lavender-blue represent clusters in China, and Japan–Korea, respectively. (c) Maximum likelihood (ML) phylogenetic tree of 171 *Q. gilva* individuals and five outgroup taxa. (d) Geographical distribution of the 35 sampled populations with color-coded groupings based on the structure analysis (*K* = 2).

The ADMIXTURE analysis based on whole-genome resequencing data revealed two clearly differentiated groups within *Q. gilva*, with the lowest cross-validation error at *K* = 2 (Supplementary Fig. S2). These groups correspond to distinct geographic distributions: a China group and a Japan–Korea group (Fig. 1a, d). Similarly, PCA separated the 35 populations into two clusters, with PC1 and PC2 explaining 11.37% and 5.89% of the total variance, respectively (Fig. 1b). A ML phylogenetic tree constructed using IQ-TREE further supported the presence of two well-resolved clades corresponding to the two geographic groups (Fig. 1c).

### Genetic diversity of *Q. gilva*

**Figure 2:**
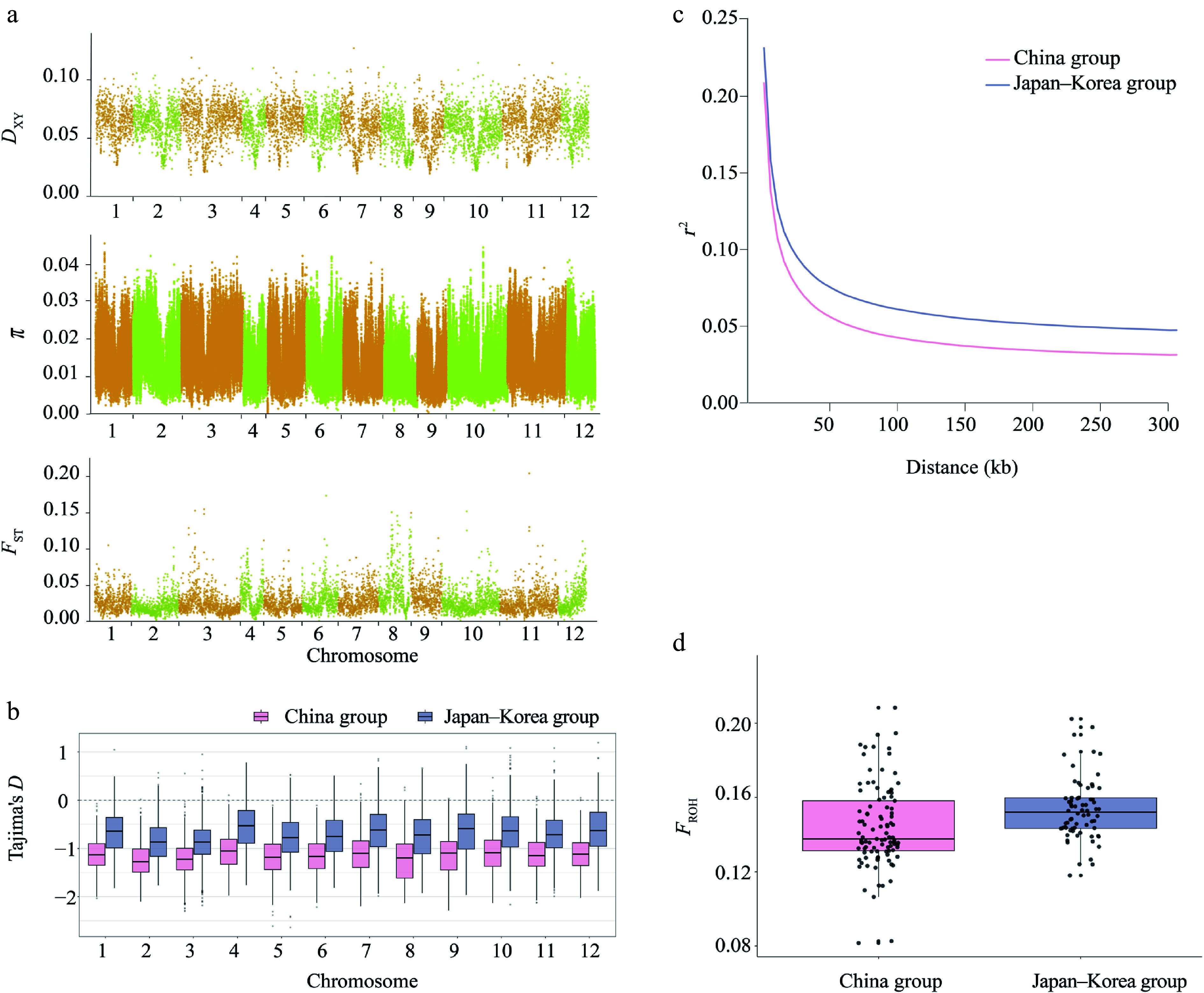
Heterogeneity in genomic diversity and genomic divergence between the China and Japan–Korea groups of *Q. gilva*. (a) Manhattan plot showing the distribution of *D*_XY_, *π*, and *F*_ST_ values between the two populations across chromosomes 1–12. The *x*-axis indicates the chromosomal position (cumulative physical position), and the *y*-axis indicates the values of *D*_XY_, *π*, and *F*_ST_. Each data point corresponds to a 100-kb sliding window. Chromosomes are alternately colored in light orange and light green to distinguish chromosome boundaries. (b) Comparison of Tajima's *D* across autosomes between two East Asian clusters. Boxplots show the distribution of Tajima's *D* values on chromosomes 1–12 for the China group (light pink) and the Japan–Korea group (lavender blue). (c) Linkage disequilibrium (LD) decay patterns in the two groups. (d) Comparison of inbreeding coefficients (*F*_ROH_) between two populations of *Q. gilva*. Light pink and lavender blue represent the China group and the Japan–Korea group, respectively.

We quantified genome-wide genetic diversity of *Q. gilva* and genomic divergence between the China and Japan–Korea groups using pixy, revealing a heterogeneous landscape across chromosomes (*π*: 0.06 × 10^−3^–4.57 × 10^−2^; *D*_XY_: 0.01862–0.1270; *F*_ST_: 0.0007–0.2049; Fig. 2a, Supplementary Fig. S3). Notably, *F*_ST_ reached 0.2049 at specific loci, indicating moderate population differentiation. Nucleotide diversity (*π*) was marginally higher in the China group (*π* = 1.41 × 10^−2^) compared to that in the Japan–Korea group (*π* = 1.39 × 10^−2^), while overall genetic differentiation between the two groups was low (*F*_ST_ = 0.028, Fig. 2a, Supplementary Table S3). Tajima's *D* values were consistently negative across all chromosomes in both groups (mean: −1.1575 in China; −0.7052 in Japan–Korea), deviating significantly from expectations under neutral evolution (Fig. 2b, Supplementary Table S3). LD decay patterns differed between groups (Fig. 2c). The maximum *r*^2^ values were 0.209 and 0.232 for the China and Japan–Korea groups, respectively. The corresponding LD decay distances (defined as the physical distance at which *r*^2^ decreases to half of its maximum value) were estimated to be approximately 10 and 15 kb, respectively (Supplementary Table S4). The Japan–Korea group exhibited stronger LD and slower decay, suggesting a more constrained recombination landscape. The inbreeding coefficient (*F*_ROH_) was calculated based on ROH longer than 10 kb across 171 individuals. ROH analysis revealed a slightly higher mean inbreeding coefficient in the Japan–Korea group compared with that in the China group (mean *F*_ROH_ = 0.153 and 0.144, respectively). In addition, the variation in the inbreeding coefficients among individuals was greater in the China group (standard deviations = 0.022 and 0.017, respectively) (Fig. 2d, Supplementary Table S5). The distribution of *F*_ROH_ values for all 35 analyzed populations is provided in the Appendix (Supplementary Fig. S4, Supplementary Table S5).

### Divergence time, demographic history, and gene flow in *Q. gilva*

**Figure 3:**
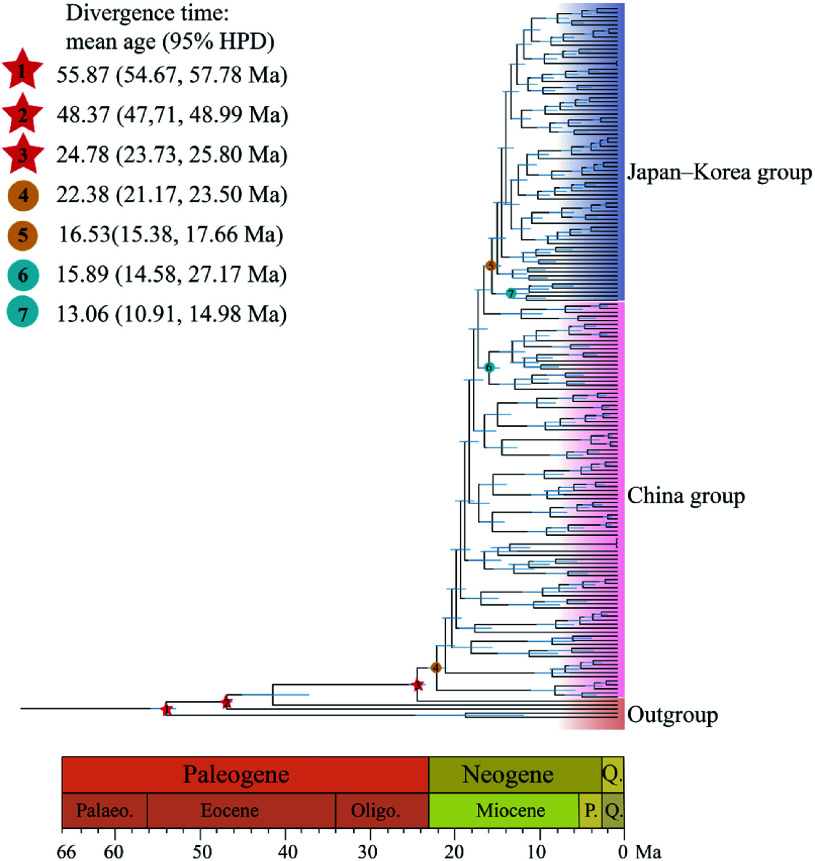
Divergence times estimated using BEAST, with blue bars indicating 95% highest posterior density (HPD) intervals. Note that red five-pointed stars indicate the three selected fossil calibrations. Geological time abbreviation: P. = Pliocene; Q. = Quaternary. Circles represent divergence time estimates for *Q. gilva* populations: (4) divergence of *Q. gilva* populations in China; (5) divergence between the China and Japan–Korea groups; (6) divergence between the Zhejiang and Taiwan populations; (7) divergence between Japanese and Korean populations.

**Figure 4:**
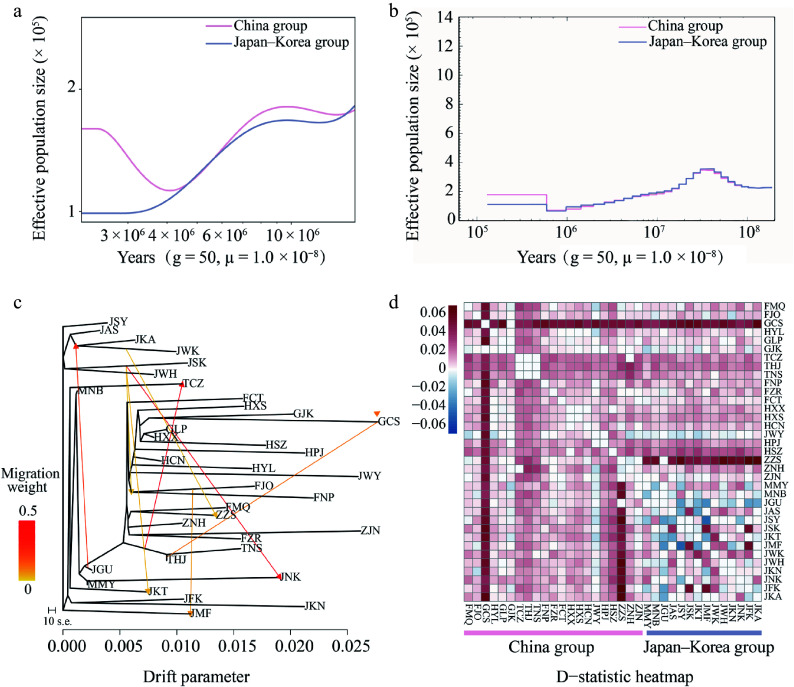
Inferred demographic histories of the China (light pink) and Japan–Korea groups (lavender-blue) based on (a) SMC++, and (b) PSMC analyses. (c) Maximum likelihood (ML) tree constructed with TreeMix, allowing for eight migration events. Migration arrows are color-scaled by weight. (d) Introgression analysis with Dsuite was presented as a heatmap, where darker shades indicate higher introgression proportions between populations. For each trio (P1, P2, P3), the D value was mapped to P2 vs. P3 as positive (+D) and to P1 vs. P3 as negative (–D).

The Bayesian divergence time Inference tree based on 171 *Q. gilva* individuals and five outgroup taxa, inferred using MCMC methods, showed a topology consistent with ML results, resolving two major clades (Figs. 1c, 3). Divergence time estimates indicate an initial split within *Q. gilva* around 24.78 Ma (95% HPD: 23.73–25.80 Ma), followed by divergence of the China group at approximately 22.38 Ma (95% HPD: 21.17–23.50 Ma), and a subsequent split between the China and Japan–Korea groups at approximately 16.53 Ma (95% HPD: 15.38–17.66 Ma) (Fig. 3). Based on the SMC++ demographic reconstruction, there is no clear evidence of a population bottleneck for *Q. gilva*. The China group experienced an effective population size drop off between the Late Miocene and Early Pliocene (from 9 Ma to 4 Ma) and recovered afterwards, whereas the Japan–Korea group exhibited a similar shrink without recovery (Fig. 4a). PSMC analyses showed synchronized expansion and contraction phases of both groups (Fig. 4b).

By integrating TreeMix and Dsuite analyses, this study systematically revealed complex historical gene flow patterns among populations. Based on model selection using the OptM package in R (Supplementary Fig. S5), an optimal TreeMix model (m = 8) was selected. This model identified multiple significant gene flow events, including the migration from JWK to ZZS (weight = 0.0360) (Fig. 4c, Supplementary Tables S6, S7). Dsuite analyses further supported widespread introgression: ZZS exchanged alleles with multiple Japanese populations, whereas GCS showed signals of introgression with all taxa except GJK. Additionally, significant gene flow was observed between TCZ and ZZS/MNB, THJ and ZZS/MNB/ZNH/FMQ, and TNS and ZNH/ZJN/FMQ (Fig. 4d, Supplementary Table S8). In summary, the optimal TreeMix model and D-statistics analyses revealed bidirectional but asymmetric gene flow between the China and Japan–Korea groups. These results not only confirm the historical genetic contributions of the China group to surrounding regions but also reveal reverse gene flow from the Japan–Korea group to the China group, reflecting the complex genetic exchange network in East Asia.

### Landscape genomic characteristics of *Q. gilva* and their vulnerability responses to future climate change

**Figure 5:**
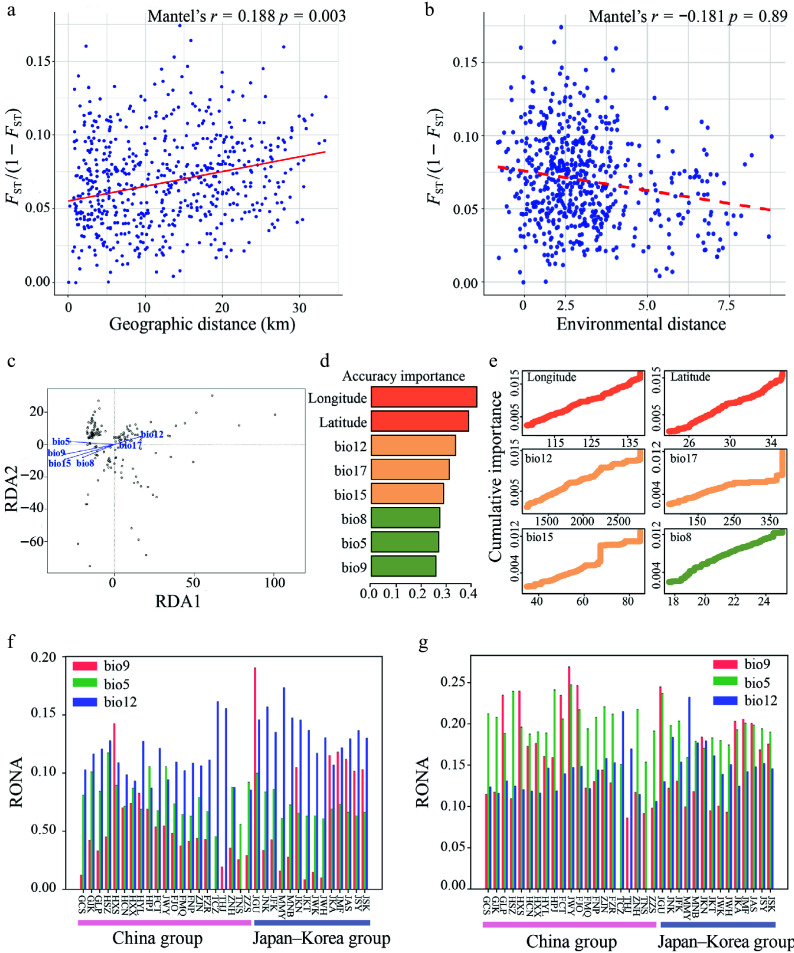
(a) Mantel test of genetic distance [*F*_ST_/(1 − *F*_ST_)] vs. geographical distance. (b) Mantel test of genetic distance [*F*_ST_/(1 − *F*_ST_)] vs. environmental distance. (c) Redundancy Analysis (RDA) of *Q. gilva* (Note that the vector length represents the contribution of each environmental variable to the explained variance, while the angles between arrows indicate correlations among variables). (d) Ranked environmental variable importance plot for *Q. gilva* based on Gradient Forest (GF) analysis. (e) I-spline curves illustrating genetic composition variation along environmental gradients. (f) Key environmental factors influencing *Q. gilva* vulnerability under SSP126 (2081−2100). (g) Key environmental factors influencing *Q. gilva* vulnerability under SSP585 (2081–2100).

The Mantel test revealed a significant correlation between genetic and geographic distances (isolation by distance (IBD): *r* = 0.188, *p* = 0.003) (Fig. 5a), but no significant correlation was observed between genetic and environmental distances (isolation by environment (IBE): *r* = –0.181, *p* = 0.89) (Fig. 5b). Several climatic variables showed significant correlations with genetic variation, including mean temperature of the wettest quarter (bio8), mean temperature of the driest quarter (bio9), annual precipitation (bio12), and precipitation seasonality (bio15) (Mantel's *r* = –0.092, *p* = 0.024; Mantel's *r* = –0.213, *p* < 0.001; Mantel's *r* = –0.118, *p* = 0.004; Mantel's *r* = –0.148, *p* < 0.001, respectively) (Supplementary Fig. S6). RDA indicated that genetic variation was significantly associated with bio5, bio8, bio9, bio12, and bio15, of which bio9 and bio15 showing the strongest effects (Proportion of Variance Explained (PVE) = 0.0093, *ρ* = 0.001) (Fig. 5c, Supplementary Table S9). We further assessed the proportion of genetic variation explained by environmental factors and geographic variables using partial redundancy analysis (pRDA) to infer the primary drivers of genetic diversity (Table 1). pRDA revealed that environmental factors (4.73%) had a significantly stronger explanatory power than that of geographic factors (1.95%). Even after controlling for geographic variables, the independent contribution of environmental factors remained substantial (4.33%), whereas that of the geographic factors was relatively weak (1.55%). This suggests that environmental filtering (e.g., climate) plays a dominant role in shaping genetic structure, whereas geographic factors (e.g., spatial distance) have a minor influence or are partially dependent on environmental variation. The full model explained only 6.28% of the total genetic variation, indicating that unmeasured factors (e.g., biotic interactions or stochastic processes) could also significantly influence the population genetic structure (Table 1).

**Table 1:** Effects of environmental and geographical factors on genetic variation decomposed using partial redundancy analysis (pRDA).

Partial RDA models	*r* ^2^	Adjusted *r*^2^	*p* Value
Combined fractions			
Full model: F~env. + F~geog.	0.0628	0.0165	0.001
F~geog.	0.0195	0.0078	0.001
F~env.	0.0473	0.0124	0.001
Individual fractions			
F~geog. | env.	0.0155	0.0041	0.001
F~env. | geog.	0.0433	0.0087	0.001
Total explained	0.0628		
Total confounded	0.004		
Total unexplained	0.9372		
Total	1		

The GF analysis identified geography as the primary driver of genetic variation in *Q. gilva*, followed by annual precipitation (bio12). Among the climatic variables, bio12, precipitation of the driest quarter (bio17), and precipitation seasonality (bio15) consistently ranked highest in importance across the *r*^2^-weighted and cumulative importance metrics. I-spline plots were generated for the top six predictors based on *r*^2^-weighted importance (Fig. 5d, e, Supplementary Fig. S7), which confirms the dominant role of these precipitation-related variables. These results highlighted the key roles of spatial structure and precipitation in shaping genetic patterns.

We assessed the genomic vulnerability of *Q. gilva* populations under future climate projections (SSP126 and SSP585, year 2081−2100) using RONA. The overall vulnerability of *Q. gilva* was higher under SSP585 (Fig. 5g, Supplementary Table S10) than under SSP126 (Fig. 5f, Supplementary Table S10). Quantification of the genetic offsets identified three climatic variables as the key drivers: annual precipitation (bio12), maximum temperature of the warmest month (bio5), and mean temperature of the driest quarter (bio9). Among these, bio12 consistently influenced the adaptive potential across all populations.

### Ecological niche modeling

**Figure 6:**
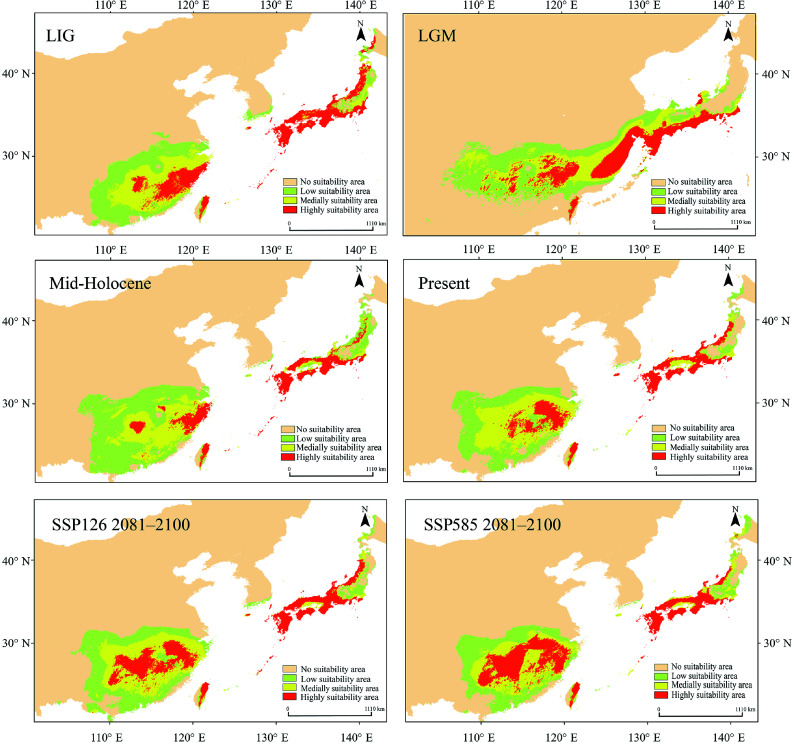
Maxent was used to simulate the ecological niches of *Q. gilva* over six time periods: Last Interglacial (LIG), Last Glacial Maximum (LGM), Mid-Holocene, present, SSP126 2081–2100, and SSP585 2081–2100. Note that the color gradient from light orange to red in the potential distribution areas represents habitat suitability levels for *Q. gilva*, ranging from unsuitable to optimal conditions.

Ecological niche modeling using MaxEnt (optimal parameters: RM = 2.5, FC = LQ) achieved high predictive accuracy (area under the curve (AUC) > 0.95; Supplementary Fig. S8, Supplementary Table S11). Projections across seven time periods revealed a dynamic distributional history. During the LGM, the exposure of land bridges facilitated an expansion of suitable habitat, which subsequently contracted during the Mid-Holocene, together displaying a unimodal trend (Fig. 6, Supplementary Table S12). Both the high- and medium-suitability areas exhibited similar temporal patterns. Future projections under SSP126 and SSP585 indicate continued range expansion, with high-suitability areas generally increasing, except for a slight decline during 2041–2060 under SSP126. Presently, suitable habitats are concentrated in subtropical East Asia, including eastern Guizhou, Hunan, Jiangxi, Fujian, Zhejiang, Taiwan, Jeju Island, and parts of Japan, which are characterized by monsoonal climates. During the LGM, the habitat contracted southward owing to glaciation, but refugia remained in the Nanling and Wuyi Mountains, Taiwan, and southern Japan. In contrast, the Mid-Holocene was characterized by northward range expansion, most notably in Japan. Under future scenarios, *Q. gilva* is predicted to expand its range, particularly into mountainous and monsoonal regions (Fig. 6, Supplementary Fig. S9).

### Adaptive potential of *Q. gilva*

**Figure 7:**
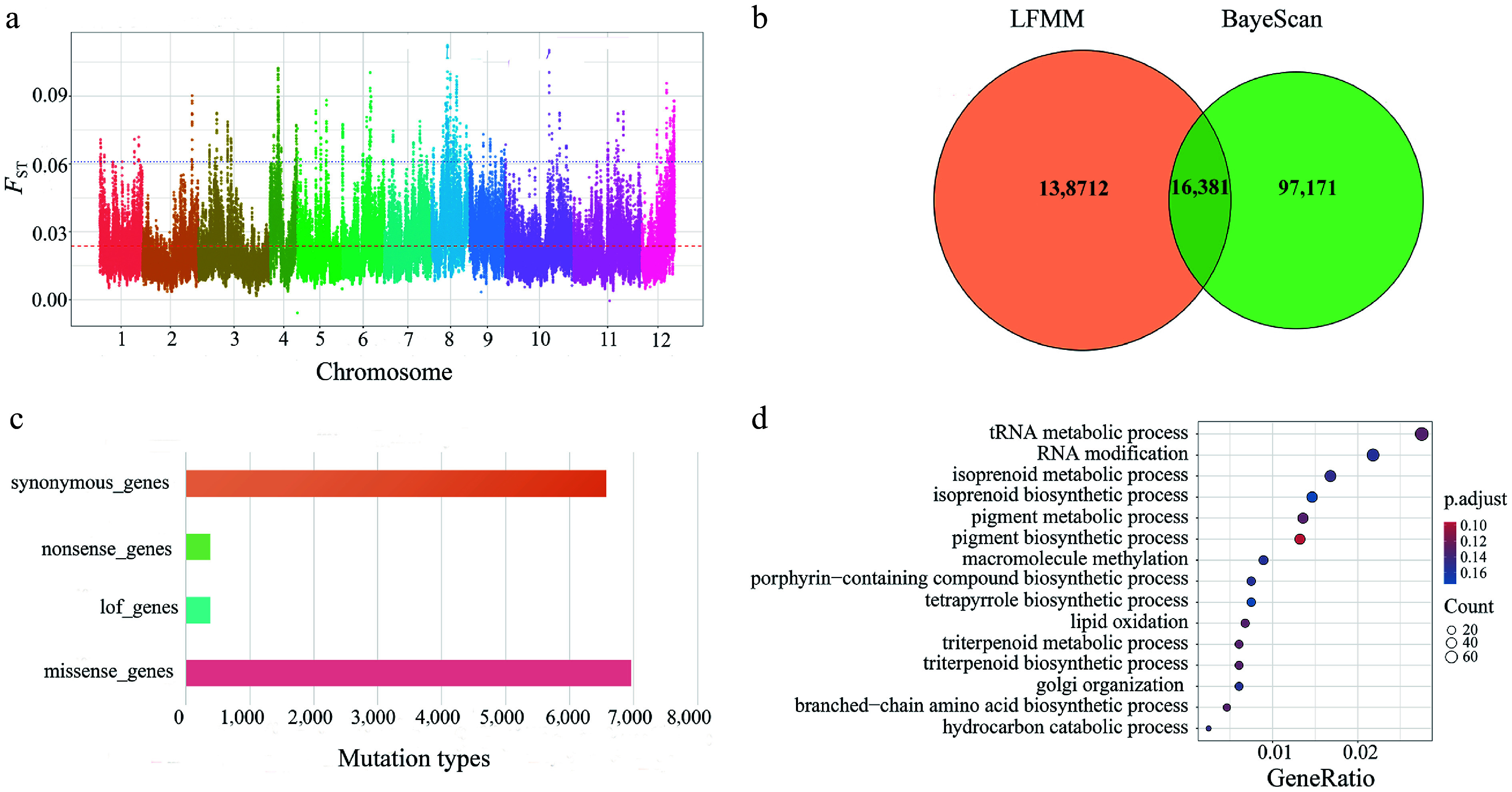
(a) Highly polymorphic loci for assessing genetic differentiation. (b) Illustrates a Venn diagram showing the detection of outlier loci based on BayeScan and LFMM. (c) Functional annotation performed using snpEff. (d) The results of GO enrichment analysis for missense mutations.

To identify the potential genetic loci underlying local adaptation between the two *Q. gilva* populations, we performed a genome-wide *F*_ST_ analysis. The Manhattan plot (Fig. 7a) revealed a heterogeneous distribution of *F*_ST_ values across the genome with prominent peaks on chromosomes 8 and 10, indicating strong population differentiation in these regions. We acknowledge that elevated *F*_ST_ values can arise from various factors, such as demographic history or genetic drift.

To more directly infer the role of natural selection, we employed two complementary genome-wide scanning approaches using the original genotype data, independent of the initial *F*_ST_ analysis: outlier detection using BayeScan to identify SNPs with allele frequency differentiation significantly exceeding neutral expectations, and environmental association analysis using LFMM to detect SNPs whose allele frequencies correlate with key climatic variables.

BayeScan identified 113,552 outlier SNPs (Supplementary Fig. S10), whereas the LFMM detected 311,414 SNPs associated with the six climatic variables (Supplementary Fig. S11). Integrative analysis revealed extensive overlap between these two sets of candidate signals across the significance thresholds. For example, at *p* ≤ 0.05, there were 277,539 environment-associated SNPs, 79,677 outlier SNPs, and 33,875 shared loci (Supplementary Fig. S12). A similar pattern was observed under more stringent thresholds: at *p* ≤ 0.01, there were 138,712 environment-associated SNPs, 97,171 outlier SNPs, and 16,381 shared loci (Fig. 7b).

The set of loci identified by both methods (e.g., the 16,381 shared SNPs at *p* ≤ 0.01) exhibit divergence that is both exceptional (outlier status) and environmentally correlated, making them high-confidence candidates for local adaptation. Based on this high-confidence candidate set (*p* ≤ 0.01, specifically the 16,381 shared loci), we performed GO enrichment analysis. The results showed that these candidate loci were significantly enriched in the core biological processes of *Q. gilva*, including disease resistance defense (e.g., salicylic acid/jasmonic acid metabolism), growth and development regulation (e.g., flowering and root system establishment), stress response (e.g., reactive oxygen species metabolism), and fundamental molecular processes (e.g., protein modification and nucleocytoplasmic transport) (Supplementary Table S13).

Functional annotation of *Q. gilva* identified four mutation types, with missense mutations being the most frequent, followed by loss-of-function, nonsense, and synonymous mutations (Fig. 7c). GO enrichment analysis of the missense mutation sites revealed their predominant involvement in plant metabolic regulation, transcriptional control, protein modification, and enzymatic activities (Fig. 7d, Supplementary Table S14).

## Discussion

The evolution of a taxon across vast spatial and temporal scales provides an unique opportunity to investigate its environmental adaptations and to address challenges posed by climate change. Population genomics has enabled a deeper understanding of the factors shaping genetic variation within and between populations under an 'Isolation-Connection-Isolation' dynamic in a well-delimited biogeographic region^[17,19,26−29]^. Landscape genomics offers insights into the genomic mechanisms underlying local adaptation and allows quantification of populations' potential maladaptation to future climate change^[5,6,73]^. Therefore, our study aimed to contextualize genome-wide genetic variation in *Q. gilva* within the framework of geography and climate change in the SJFR of the EBLFs.

Using 35 natural populations spanning the entire distribution range of *Q. gilva*, we demonstrate that this species exhibits a consistent biogeographic break across the East China Sea (ECS), corresponding to the phylogeographic differentiation between the China and Japan–Korea groups^[17,19,20,23−27]^. Although this pattern has been identified in several taxa using first- and second-generation sequencing data, substantial discrepancies persist in the estimated divergence times^[19,74]^. Both geographic and environmental gradients jointly shaped the differentiation of *Q. gilva* into these two groups. Molecular evidence strongly supports the environmental adaptation hypothesis of this species, indicating that precipitation exerts a significantly stronger selective pressure than temperature.

### Geographical origin, divergence, and demographic history of *Q. gilva*

#### Miocene origin and eastward expansion

East Asian subtropical EBLFs developed concomitantly with Asian monsoon systems and occurred no earlier than the Eocene^[17,75,76]^. The current EBLFs harbor an unique assemblage comprising boreotropical relics, tropical flora, and deciduous broad-leaved forests^[17,19,20,23−27,32,77]^. Our study suggests that *Q. gilva* initially diversified in southwestern China, followed by eastward dispersal to eastern China and Japan, driven by an enhanced East Asian monsoon during the Early to Middle Miocene. We also dated the divergence between the China and Japan–Korea groups between the late Early Miocene and the Early Mid-Miocene. The Japanese archipelago began to separate from the Eurasian continent approximately 24 Ma^[19]^. This indicates that a corridor across the ECS existed for species dispersal between East Asia and Japan during the early Mid-Miocene. Our newly estimated divergence time for this differentiation coincides with increasingly colder and arid climates following the Mid-Miocene Climatic Optimum (ca. 17–15 Ma)^[78,79]^. Consistent with previous studies, *Q. gilva* dispersed eastward, eventually reaching the Japanese archipelago via the Zhoushan region of Zhejiang Province^[25,80,81]^. This migration pathway showed remarkable congruence with the endemic distribution patterns observed in the biodiversity hotspot regions of eastern China and southern Japan.

#### Quaternary refugia and land bridge-mediated gene flow

It has been proposed that the continental shelf of the ECS reemerged as a corridor for species migration during the LGM^[18]^. Recently, phylogeographic studies on two Sino-Japanese disjunct species (*Neolitsea sericea* and *Machilus thunbergii*) found genetic signals of lineage admixture on either side of the ECS^[27,82]^. One of the most significant discoveries of this study was the detection of two admixed genetic groups coexisting in the populations from Zhoushan, Kyushu, and southwestern Honshu. This pattern provides strong evidence that the ECS land bridge facilitated secondary contact between these groups during the glacial periods. Meanwhile, the minimal differentiation between the Japanese and Korean populations indicated substantial historical gene flow (Supplementary Table S15).

Ecological niche modeling and palynological records demonstrated that *Q. gilva* responded to climatic cooling during the LGM through southward migration, with southern Japan and Jeju Island serving as key refugia^[83,84]^. The 'Sino-Japanese admixed' genetic characteristics of Korean populations, combined with geological evidence of the Tsushima land bridge during glacial periods, suggest their postglacial recolonization of the Korean Peninsula from Japanese refugia^[85,86]^. Meanwhile, the land bridge across the Taiwan Strait, formed during glacial periods due to lowered sea levels, provided migration corridors for multiple species from mainland China to Taiwan^[19,87−90]^. Recurring land connections during the Quaternary glacial–interglacial cycles maintained the gene flow of populations on the mainland and in Taiwan. Notably, based on molecular evidence, we found that the Taiwanese *Q. gilva* population (TCZ) exhibited a complex genetic structure—its genetic composition represented an admixture of mainland Chinese and Japanese–Korean groups. Similar biogeographic patterns have been documented in multiple East Asian woody plant taxa (e.g., *Quercus* and *Camphora*)^[91,92]^, providing compelling evidence for the pivotal role of glacial land bridges in shaping regional biogeographic patterns. The phylogenetic affinities and biogeographic connections of flora among Taiwan of China, mainland of China, and Japan may have been established via multiple pathways: (1) land-bridge connections via the exposed ECS continental shelf during glacial periods; (2) stepping-stone dispersal through the Ryukyu Archipelago; and (3) anthropogenic introductions^[76,93−95]^.

The phylogeographic history of *Q. gilva* reconstructed in the present study provides a clear example for species evolution of East Asia subtropical EBLFs. Combining this and other studies demonstrates that the contemporary distribution patterns of EBLFs are the products of intertwined long-term natural evolutionary processes (climatic fluctuations and geological events) and recent anthropogenic influences^[77,96−98]^. This integrated perspective not only enhances our understanding of how organisms respond to past environmental changes, but also provides a critical framework for predicting and managing their responses to ongoing and future climate change.

#### Evolutionary trajectory and demographic history shaped the genetic diversity of Q. gilva

Genetic diversity underpins species' adaptation to environmental changes and their long-term evolutionary potential, and is therefore recognized as a central objective of biodiversity conservation^[99,100]^. Although *Q. gilva* is currently assessed as endangered^[32]^, its genome-wide nucleotide diversity (*π* ≈ 1.4 × 10^−2^) unexpectedly exceeds that of its widespread congeners, including *Q. acutissima*, *Q. variabilis*, and *Q. dentata* (*π* = 0.87 – 0.95 × 10^−^^2^)^[101,102]^. Long-lived organisms such as trees exhibit remarkable resilience in their effective population size over millions of years of glacial cycles, buffering genetic diversity against climatic fluctuations^[103,104]^. Our demographic reconstructions support the view that *Q. gilva* has experienced no severe genetic bottlenecks throughout its long evolutionary history, which likely explains its relatively high genetic diversity. In contrast to species that suffer from strong historical bottlenecks^[105]^, populations of *Q. gilva* may have survived glacial periods in multiple microrefugia, maintaining allelic diversity through postglacial secondary contact^[106]^. Furthermore, our RONA analysis (consistently below 0.25) indicated a low mismatch between the genetic composition and future environments, suggesting a robust adaptive capacity. Notably, adaptive introgression is widespread in the genus *Quercus*, and interspecific gene flow may have also contributed to the maintenance of its genetic diversity^[101,107]^. Thus, the genetic diversity pattern of *Q. gilva* may benefit from the interplay between its long evolutionary history and complex population dynamics. However, the stark contrast between its high evolutionary potential and its current endangered status underscores the profound impact of recent anthropogenic activities. Deforestation and habitat destruction have caused rapid local population extinctions and severe fragmentation of extant populations^[32]^. Thus, this study indicates that although long-term evolutionary history has endowed species with a profound adaptive capacity, short-term, high-intensity anthropogenic disturbances can still rapidly drive them toward extinction^[108]^.

The subtle but consistent intraspecific difference, where the China group retains slightly higher diversity than the Japan–Korea group (Fig. 2a, Supplementary Table S3), further illuminates our understanding of its biogeographic history. This implies that despite the overarching pattern of high diversity, the complex topography of mainland China (e.g., the interconnected mountain ranges of the Yunnan-Guizhou Plateau and the Wuyi Mountains) may have provided a more stable and connected glacial refugia, better preserving genetic variation^[32]^. In contrast, the insular and peninsular nature of the Japan–Korea range likely imposed a greater isolation and bottleneck effect^[32,109,110]^.

Overall, the genetic structure of *Q. gilva* revealed a significant genetic affinity between the China and Japan–Korea groups of *Q. gilva*. This close genetic connection across geographically isolated populations may provide an evolutionary explanation for the species' biogeographic distribution pattern. Further analyses demonstrated that the complex genetic diversity patterns in *Q. gilva* essentially reflect the dynamic interactions between geological changes and ecological processes, which collectively shape the diversified distribution and adaptive capacity of the species across East Asian landscapes.

### Genetic variation of *Q. gilva* in response to environmental gradients and its climate adaptation potential

Under climate change scenarios, understanding the genetic basis of species' adaptation and evaluating their potential to respond to future climates is critical^[111]^. Previous studies have employed diverse strategies to reveal the survival risks faced by species under climate change^[112,113]^. Mantel tests revealed a significant correlation between genetic differentiation and geographic distance in *Q. gilva* populations. This pattern reflects the species' limited dispersal capacity: as a wind-pollinated species producing single-seeded nuts with hard pericarps, which are primarily dispersed by small rodents (e.g., squirrels and jays) through scatter-hoarding behavior^[114]^, and with prolonged germination periods and low germination rates^[115,116]^, *Q. gilva* exhibits stronger gene flow among neighboring populations and limited exchange between distant ones. Landscape genomic analyses demonstrated that both precipitation and temperature drive adaptive genetic variation in *Q. gilva*, with precipitation exerting a significantly stronger selective pressure than temperature (Fig. 5c−e). Notably, the relative importance of these climatic pressures exhibits distinct regional differentiation among *Quercus* species: genome-wide association studies have identified adaptive SNPs in European populations of *Q. pubescens* and *Q. robur*, primarily associated with precipitation^[117]^, whereas North American populations of *Q. petraea* and *Q. lobata* showed stronger genetic responses to temperature gradients^[118]^. These interspecific differences likely reflect the adaptive divergence strategies of *Quercus* species in response to distinct regional climates^[119]^. As a pivotal tectonic driver, the uplift of the Tibetan Plateau since the Miocene dramatically intensified the East Asian monsoon system^[19,80,106]^, thereby shaping unique hydrothermal regimes across East Asia. This geohistorical process provides a critical environmental context for understanding the stronger adaptive response to precipitation observed in East Asian endemic species such as *Q. gilva*.

Despite this historical adaptive legacy, our study reveals a critical conservation dilemma. Ecological niche modeling projects an expansion of climatically suitable habitats for *Q. gilva* across East Asia, especially in montane- and monsoon-affected regions^[15]^. However, this optimistic projection is challenged by the high genomic vulnerability observed in the Japan–Korea group (Supplementary Table S10), indicating a constrained capacity to adapt to climate change. Consequently, even in future suitable habitats, these rear-edge populations may face elevated extinction risks, resulting from a mismatch between environmental opportunities and evolutionary potential^[12]^.

The molecular evidence strongly supports the environmental adaptation hypothesis of *Q. gilva*. Genome-wide analyses have revealed significant enrichment of multiple stress-resistance-related functional modules^[120−122]^. Regarding ion homeostasis regulation, the enrichment of potassium channels (e.g., *AKT1*) and calcium signaling pathways (GO:0022890) demonstrates their close association with plant salt tolerance, which enhances stress resistance through regulation of the SOS pathway^[123,124]^. In the stress response regulatory network, the WRKY transcription factor family (GO:0045893) and SnRK2 protein kinases (GO:0043687) serve as key regulators that improve drought resistance by modulating antioxidant gene expression and participating in ABA signaling pathways, respectively^[125,126]^. In terms of metabolic adaptation, sugar metabolism pathways (GO:1901137) and trehalose biosynthesis enhance the adaptive capacity by accumulating osmoregulatory substances and providing thermotolerance protection^[127−129]^. These findings are highly consistent with recent studies on adaptive evolution in woody plants^[130,131]^ and support the 'gene network reprogramming' hypothesis^[132]^, indicating that *Q. gilva* achieves environmental adaptation through multi-layered regulatory mechanisms. Future research should validate the functions of key genes (e.g., trehalose-6-phosphate synthase) and their specific roles in stress resistance^[133,134]^.

Therefore, by evaluating how environmental heterogeneity shapes adaptive variation in *Q. gilva* and reveals its genomic vulnerability, we have addressed the second major focus of this study. Our findings not only highlight species-specific adaptation to the East Asian monsoon, but also caution against over-optimistic projections based solely on habitat suitability^[135]^.

## Conclusions

This study establishes, for the first time, an evolutionary history and environmental adaptation framework for the East Asian evergreen broad-leaved tree species *Q. gilva*. Our findings reveal that its biogeographic expansion was jointly driven by the intensification of the Miocene monsoon and the dynamic emergence of land bridges during glacial-interglacial cycles, resulting in divergence between the China and Japan–Korea groups and their subsequent periodic secondary contact with hybrid distribution patterns. We identified annual precipitation as the dominant climatic factor determining its distribution pattern, highlighting species-specific adaptations to precipitation variability in the East Asian monsoon region. These findings not only elucidate the genetic resilience mechanisms of widespread East Asian evergreen broad-leaved tree species during geological and climatic upheavals, but also provide a theoretical baseline for predicting their responses to future climate change. The multidisciplinary integration paradigm proposed in this study offers an universal template for analyzing the eco-evolutionary dynamics of other widely distributed species in monsoon regions.

## SUPPLEMENTARY DATA

Supplementary data to this article can be found online.

## Data Availability

All data have been deposited at the Genome Sequence Archive at the National Genomics Data Center, China National Center for Bioinformation, under accession number CRA028865.
