# Supplementary material for: Spatiotemporal genomic patterns of Quercus gilva: decoupling historical isolation from contemporary environmental adaptation
Source: For Res (Fayettev). 2026 Apr 28;6:e016. doi: 10.48130/forres-0026-0016 (PMC13195491; doi:10.48130/forres-0026-0016)
Supplement: Supplementary file 1 — Supplementary data to this article can be found online. [file forres-0026-0016-S1.zip › 10.48130_forres-0026-0016-Suppl-FigureS1.pdf]

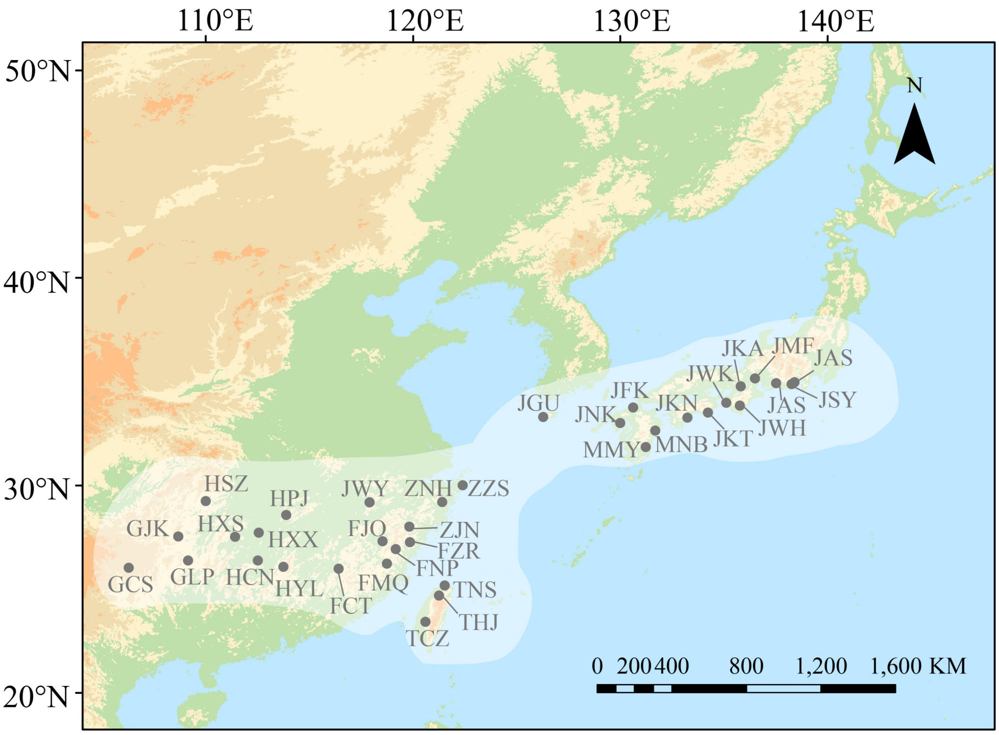

Supplementary Fig. S1 The geographical distribution of *Quercus gilva*. Black dots on the map represent our field collection sites.
