# Supplementary material for: Spatiotemporal genomic patterns of Quercus gilva: decoupling historical isolation from contemporary environmental adaptation
Source: For Res (Fayettev). 2026 Apr 28;6:e016. doi: 10.48130/forres-0026-0016 (PMC13195491; doi:10.48130/forres-0026-0016)
Supplement: Supplementary file 1 — Supplementary data to this article can be found online. [file forres-0026-0016-S1.zip › 10.48130_forres-0026-0016-Suppl-FigureS11.pdf]

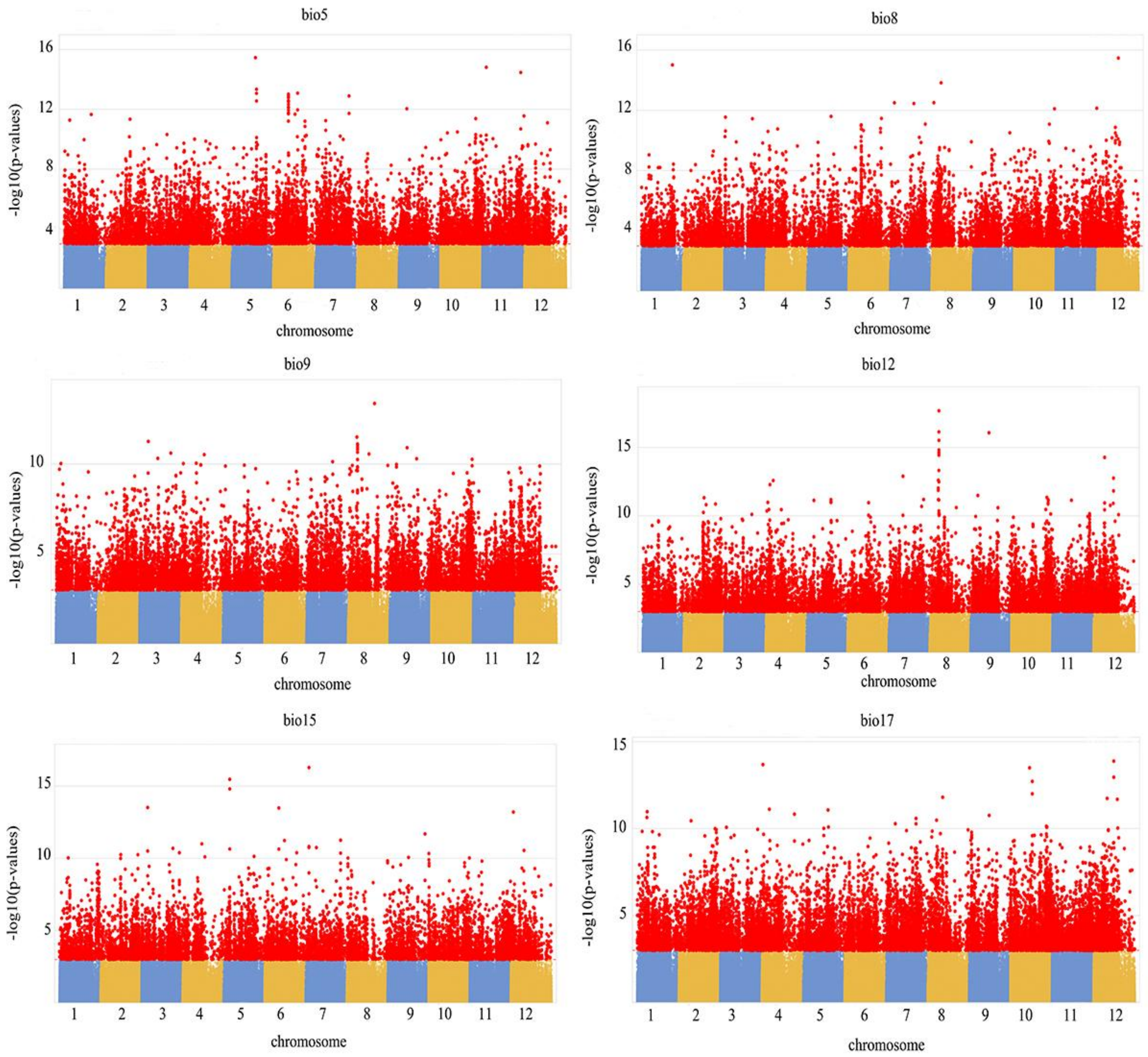

Supplementary Fig. S11 Loci screened based on LFMM. Gray and orange points represent all tested SNPs, while red points indicate outlier SNPs with significant associations (LFMM, p<0.001). The X-axis shows the genomic position of SNPs along the chromosomes, and the Y-axis displays the negative logarithm of the association p-value ($-\log_{10}(p\text{-value})$). The dashed line marks the statistical significance threshold ($-\log_{10}(0.001)=3$).
