# Supplementary material for: Spatiotemporal genomic patterns of Quercus gilva: decoupling historical isolation from contemporary environmental adaptation
Source: For Res (Fayettev). 2026 Apr 28;6:e016. doi: 10.48130/forres-0026-0016 (PMC13195491; doi:10.48130/forres-0026-0016)
Supplement: Supplementary file 1 — Supplementary data to this article can be found online. [file forres-0026-0016-S1.zip › 10.48130_forres-0026-0016-Suppl-FigureS3.pdf]

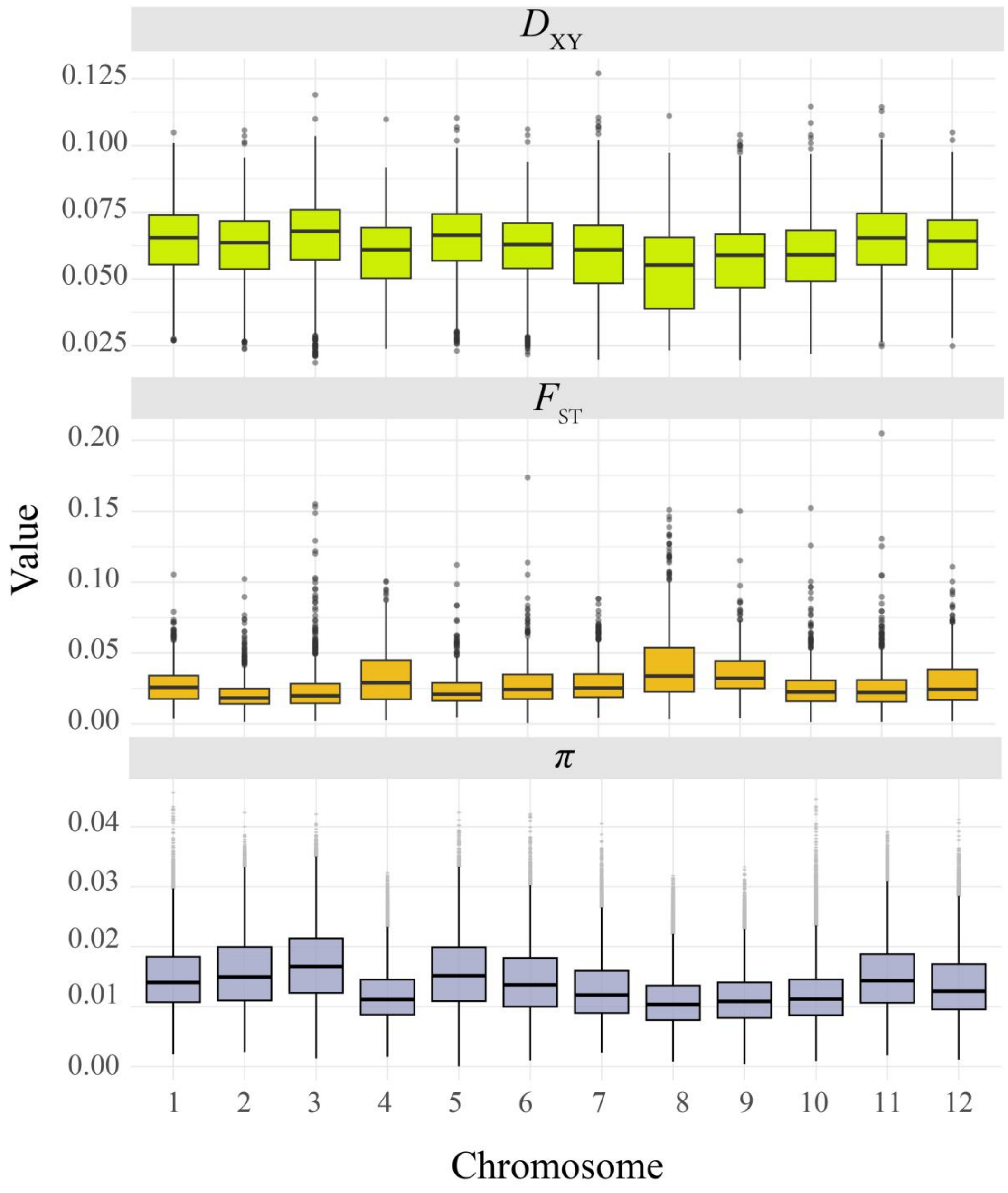

Supplementary Fig. S3 Combined box plots display the distribution of three population genetics statistics (D_{XY}: between-population genetic differentiation; π: nucleotide diversity; F_{ST}: population differentiation coefficient) across chromosomes 1–12. Each panel corresponds to a single statistic, and each box represents the value distribution for one chromosome, showing the median (inner horizontal line), interquartile range (box), and outliers (points outside the box). The y-axis scale is set independently according to the value range of each statistic. D_{XY} and π are absolute values, while F_{ST} is a relative value ranging from 0 to 1. This plot can be used to compare patterns of genetic diversity and differentiation across different chromosomes.
