# Supplementary material for: Spatiotemporal genomic patterns of Quercus gilva: decoupling historical isolation from contemporary environmental adaptation
Source: For Res (Fayettev). 2026 Apr 28;6:e016. doi: 10.48130/forres-0026-0016 (PMC13195491; doi:10.48130/forres-0026-0016)
Supplement: Supplementary file 1 — Supplementary data to this article can be found online. [file forres-0026-0016-S1.zip › 10.48130_forres-0026-0016-Suppl-FigureS4.pdf]

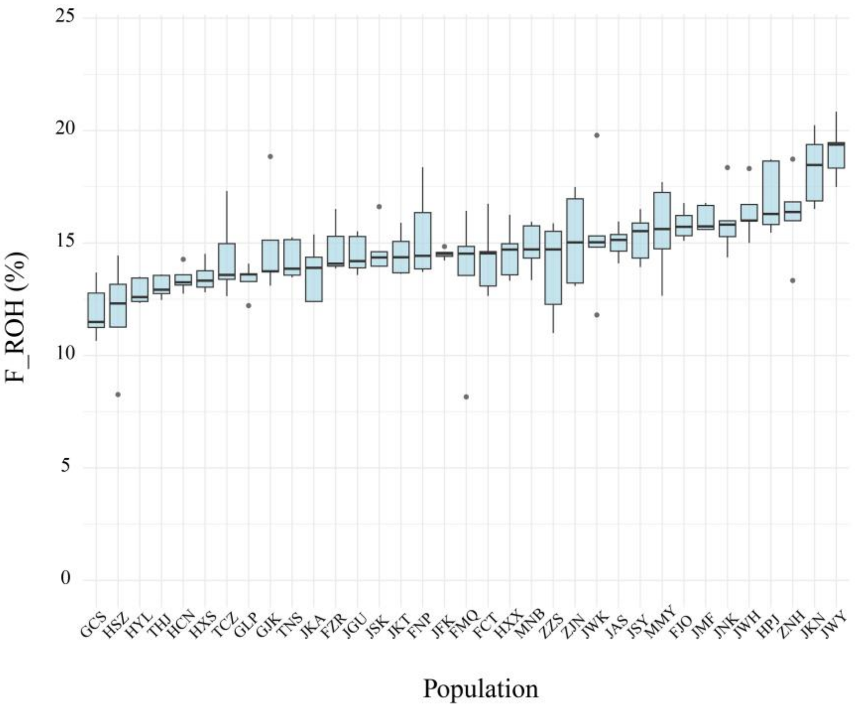

Supplementary Fig. S4 Distribution of the inbreeding coefficient (F_{ROH}) across 35 populations. The inbreeding coefficient (F_{ROH}, expressed as a percentage) was calculated based on runs of homozygosity (ROH) longer than 10 kb. The box represents the interquartile range (IQR; 25th–75th percentiles), the horizontal line inside the box indicates the median, whiskers extend to 1.5 × IQR, and individual data points are plotted as dots.
