# Supplementary material for: Spatiotemporal genomic patterns of Quercus gilva: decoupling historical isolation from contemporary environmental adaptation
Source: For Res (Fayettev). 2026 Apr 28;6:e016. doi: 10.48130/forres-0026-0016 (PMC13195491; doi:10.48130/forres-0026-0016)
Supplement: Supplementary file 1 — Supplementary data to this article can be found online. [file forres-0026-0016-S1.zip › 10.48130_forres-0026-0016-Suppl-FigureS9.pdf]

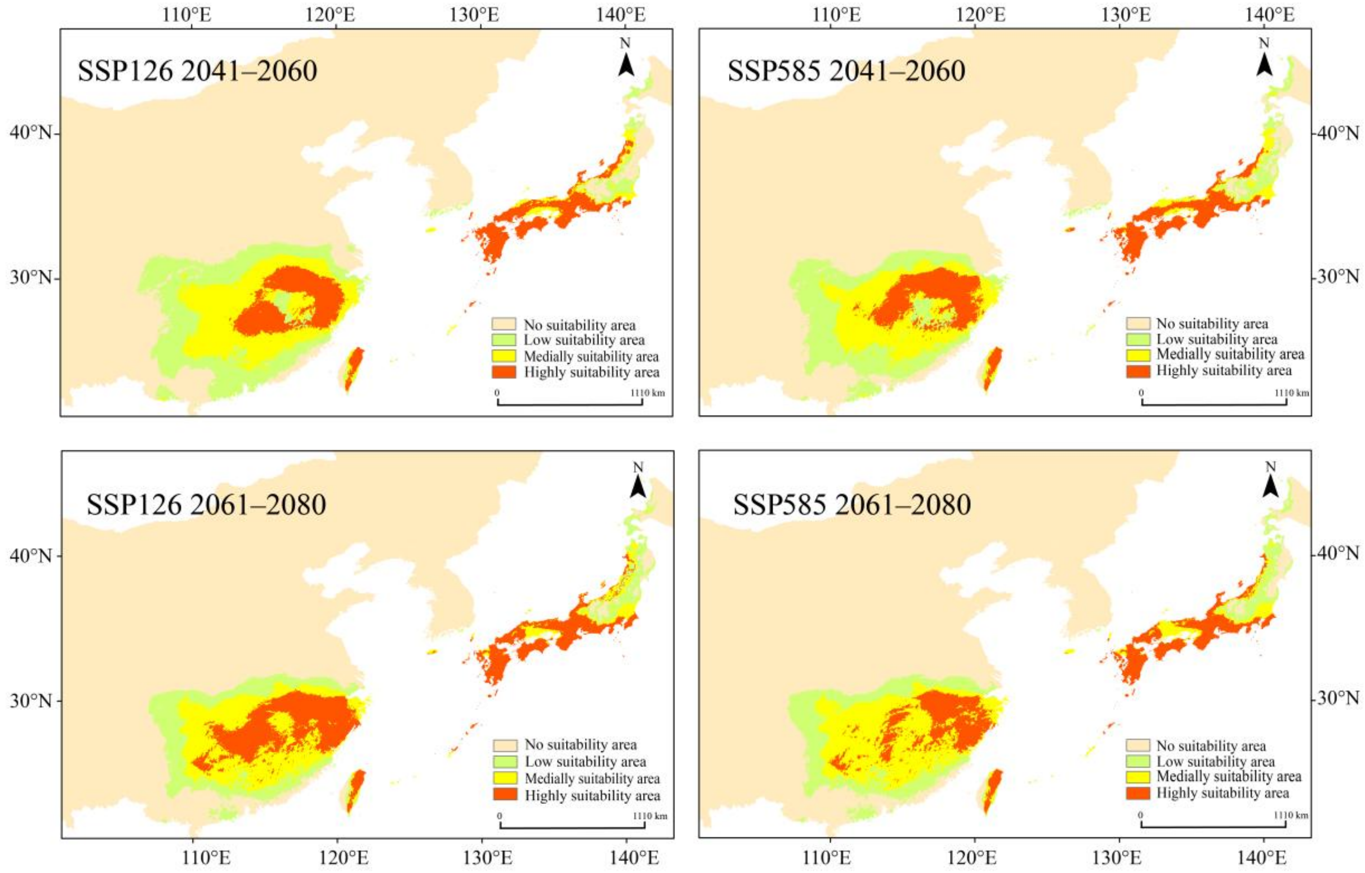

Supplementary Fig. S9 Simulation of ecological niches of *Q. gilva* using Maxent across four time periods: SSP126 2041–2060, SSP585 2041–2060, SSP126 2061–2080, and SSP585 2061–2080.
