# Supplementary figures and images for: Spatiotemporal genomic patterns of Quercus gilva: decoupling historical isolation from contemporary environmental adaptation
Source: For Res (Fayettev). 2026 Apr 28;6:e016. doi: 10.48130/forres-0026-0016 (PMC13195491; doi:10.48130/forres-0026-0016)

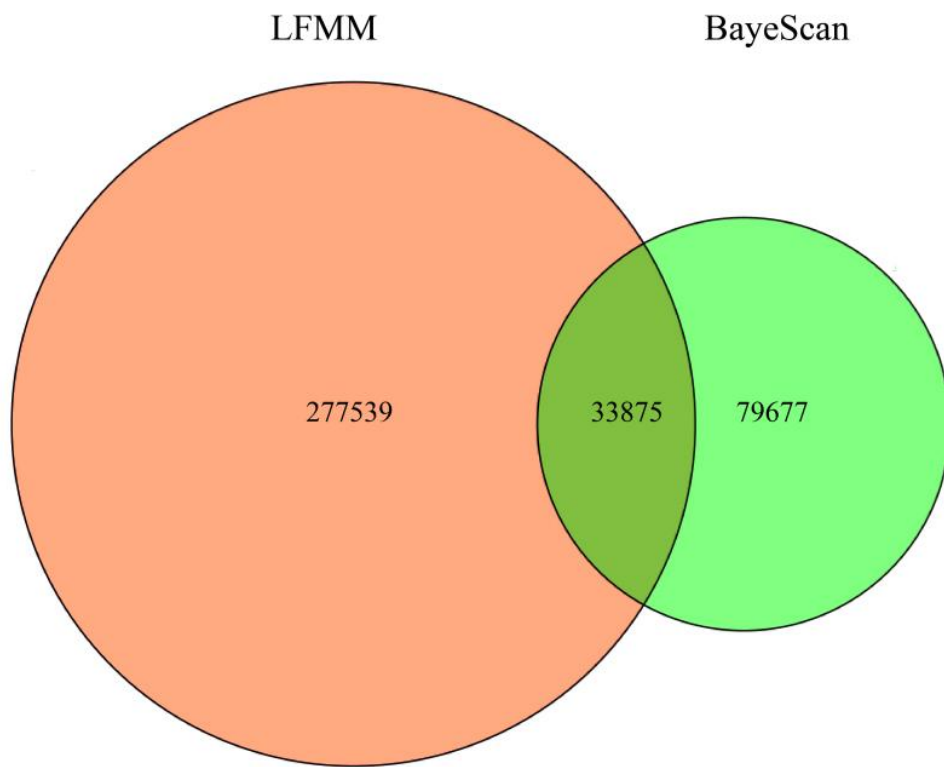

**Supplementary Fig. S12** Venn diagram of outlier loci detected by BayeScan and LFMM ( $p \leq 0.05$ ).

Supplement: Supplementary file 1 — Supplementary data to this article can be found online. [file forres-0026-0016-S1.zip › 10.48130_forres-0026-0016-Suppl-FigureS12.pdf]

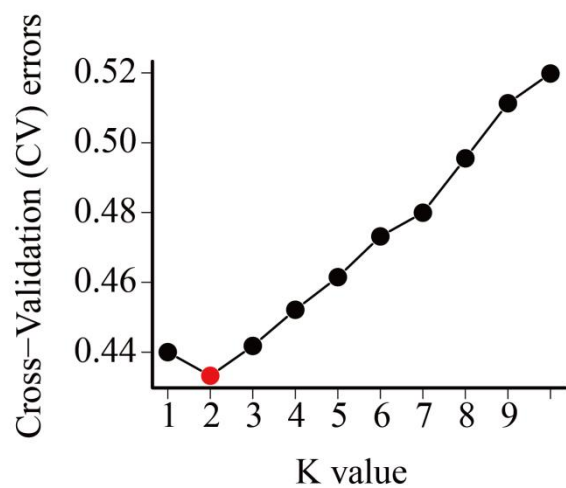

**Supplementary Fig. S2** Cross-validation error (CV) values from ADMIXTURE analysis.

Supplement: Supplementary file 1 — Supplementary data to this article can be found online. [file forres-0026-0016-S1.zip › 10.48130_forres-0026-0016-Suppl-FigureS2.pdf]

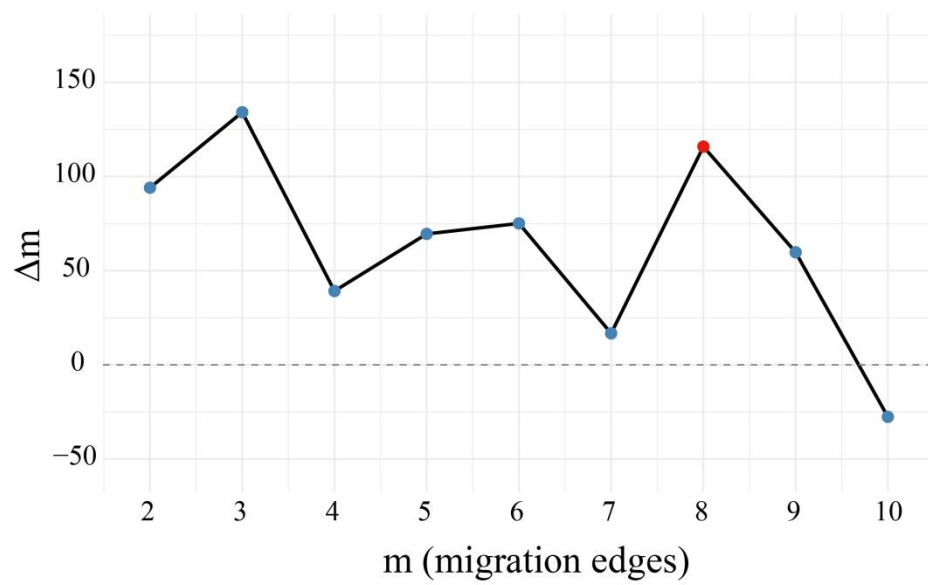

**Supplementary Fig. S5** Distribution of  $\Delta m$  inferred by OptM.

Supplement: Supplementary file 1 — Supplementary data to this article can be found online. [file forres-0026-0016-S1.zip › 10.48130_forres-0026-0016-Suppl-FigureS5.pdf]

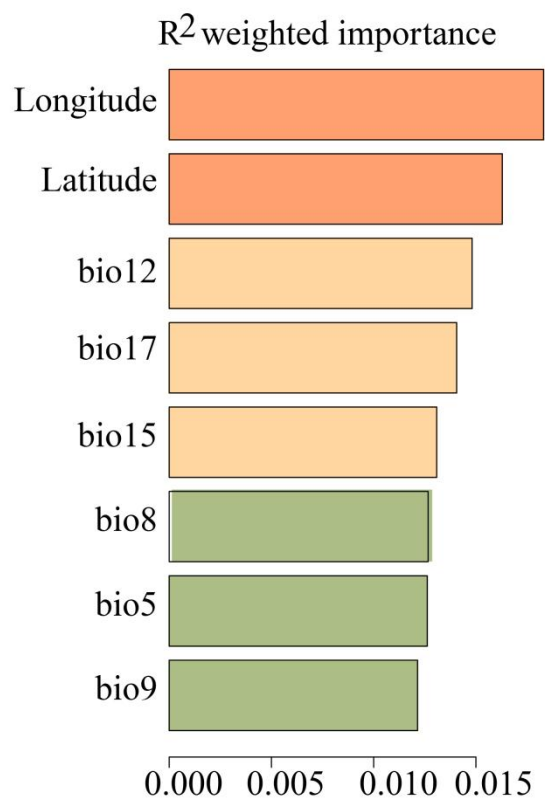

**Supplementary Fig. S7** The R<sup>2</sup>-weighted importance metric in gradient forests.

Supplement: Supplementary file 1 — Supplementary data to this article can be found online. [file forres-0026-0016-S1.zip › 10.48130_forres-0026-0016-Suppl-FigureS7.pdf]

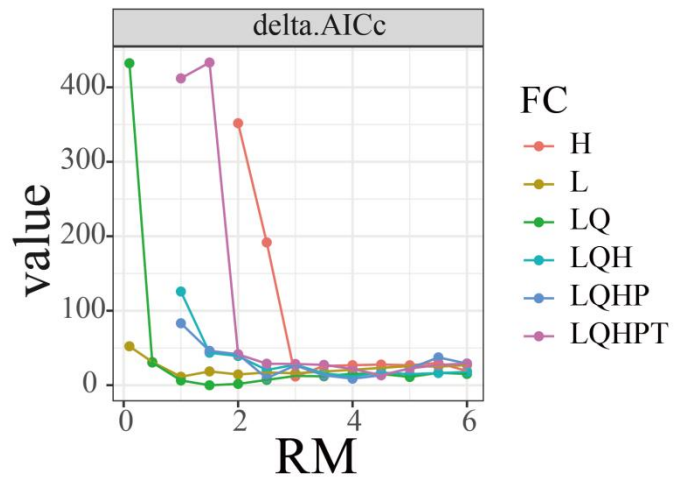

**Supplementary Fig. S8** Optimal model parameters.

Supplement: Supplementary file 1 — Supplementary data to this article can be found online. [file forres-0026-0016-S1.zip › 10.48130_forres-0026-0016-Suppl-FigureS8.pdf]
